# Highlights of New Strategies to Increase the Efficacy of Transition Metal Complexes for Cancer Treatments

**DOI:** 10.3390/molecules28010273

**Published:** 2022-12-29

**Authors:** Ester Giorgi, Francesca Binacchi, Carlo Marotta, Damiano Cirri, Chiara Gabbiani, Alessandro Pratesi

**Affiliations:** Department of Chemistry and Industrial Chemistry, University of Pisa, Via G. Moruzzi 13, 56124 Pisa, Italy

**Keywords:** platinum compounds, gold compounds, metal-based drugs, heterobimetallic complexes, targeting strategies

## Abstract

Although important progress has been made, cancer still remains a complex disease to treat. Serious side effects, the insurgence of resistance and poor selectivity are some of the problems associated with the classical metal-based anti-cancer therapies currently in clinical use. New treatment approaches are still needed to increase cancer patient survival without cancer recurrence. Herein, we reviewed two promising—at least in our opinion—new strategies to increase the efficacy of transition metal-based complexes. First, we considered the possibility of assembling two biologically active fragments containing different metal centres into the same molecule, thus obtaining a heterobimetallic complex. A critical comparison with the monometallic counterparts was done. The reviewed literature has been divided into two groups: the case of platinum; the case of gold. Secondly, the conjugation of metal-based complexes to a targeting moiety was discussed. Particularly, we highlighted some interesting examples of compounds targeting cancer cell organelles according to a third-order targeting approach, and complexes targeting the whole cancer cell, according to a second-order targeting strategy.

## 1. Introduction

During the last decades, great strides have been made in the fight against cancer. The knowledge about the mechanisms of its onset and cellular resistance has notably increased, as well as the discovery of new potential therapies. Despite this, cancer remains a problematic pathology to treat due to its characteristics of aggression, ability to metastasize, resistance onset, and severe side effects of the available drugs. For these reasons, cancer is still the second leading cause of morbidity and mortality worldwide [1]. In the context of the most promising anticancer therapies developed so far, metal-based drugs have represented for several decades the cornerstone for tumour chemotherapy [2,3]. In fact, platinum complexes, such as cisplatin, oxaliplatin, and carboplatin, are still the most widely used treatments in several kinds of cancer, including testicular, cervical, ovarian, and non-small cell lung cancers. However, in several cases after a variable number of chemotherapy cycles with platinum-based complexes, tumoral cells could become less susceptible to the cytotoxic effects of the drug, thanks to the activation of a multifactorial adaptive response that involves three major components: autophagy, endoplasmic reticulum stress signalling, and senescence [4]. Thus, the majority of cisplatin-treated patients experience therapeutic failure and tumour recurrence during—or right after—the treatments [5]. In addition, there are severe side effects associated with therapeutic regimens based on platinum complexes, such as nephrotoxicity, neurotoxicity, leukopenia, thrombocytopenia, gastrointestinal issues, nausea, vomiting, and hair loss [6]. These latter are mainly due to poor drug selectivity causing the death of healthy cells together with the tumoral ones. Considering all these issues, improvements in developing new effective therapies can indeed be pursued. These problems have prompted researchers to focus on developing a new generation of chemotherapeutics. Firstly, the research has been extended to other transition metal compounds, such as gold, ruthenium, silver, iridium, rhenium, and copper, that proved to have important cytotoxic effects on cancer cells, becoming promising potential new anticancer drugs. For example, the gold compounds’ popularity increased following the idea to repurpose Auranofin, an Au(I)-based complex approved by the FDA in 1985 as an antirheumatic Au(I) agent, for the treatment of cancer [7]. In fact, several studies highlighted its promising cytotoxic properties, entering also in three separate clinical trials in the United States as an anticancer compound [8]. Additionally, for Ru-based compounds the interest of the scientific community significantly grew after the publication of NAMI-A, KP1019 and KP1339, which are three Ru(III) complexes that have entered other human clinical trials [9]. During the past years, various strategies have been explored to optimise the efficiency of metal complexes with anti-cancer activity, both in potency and selectivity, aiming to obtain the so coveted “*anticancer magic bullet*”, already conceptualised in the early 1900s by Paul Ehrlich [10]. In this review, we will focus on some examples referring to two main strategies: the first one regards heterobimetallic complexes with a synergic action, while the second one refers to constructs where the metallic complex was conjugated to a targeting moiety. Starting from the promising results obtained with some monometallic complexes, a new approach was attempted combining different metal centres in one compound by forming a heterobimetallic complex able to combine the various modes of action that are typical of the constituting metal centres. The heterobimetallic compound should enhance the anticancer properties of single metallodrugs. The objective is to enlighten any possible synergistic effect due to the administration of two or more active ingredients as a single drug [11,12]. Generally, the two metal centres have a different final molecular target with diverse mechanisms of action, or they can modify different pathways in the cellular metabolism, or adjust some chemical-physical properties of the whole heterobimetallic complex, such as the solubility or lipophilicity, with different contributions [13]. The combination of different activities into one single drug should reduce the concentration needed for the therapeutic approach, and this should decrease the side effects produced by a large amount of compound needed to have a positive response to the therapy. This behaviour could be expressed in lower IC_50_ values or by overcoming the resistant problems to the current therapies. Another appealing approach involves targeting strategies, which has proved to have many advantages and potential interesting applications. Targeted anticancer agents are designed according to a strategy that aims to direct them towards cancer-specific biomarkers or biological targets which are overexpressed in cancer cells. The aim is to achieve higher selectivity than standard platinum-based therapies and, consequently, lower side effects. In fact, preferential recruitment of the drug into tumour cells results in a selective cytotoxic action that damages tumour cells more than healthy ones. After being preferentially accumulated in tumour cells, the drug interacts with specific targets by modifying or interrupting a signalling pathway that supports the evasion of apoptosis, tumour growth, and resistance in cancer cells. While in classical therapies the focus is on discovering increasingly cytotoxic and potent drugs, in targeted therapies the aim is to achieve selectivity on diseased cells, potentially leading to more significant and controlled cytotoxicity [14].

## 2. Heteronuclear Metal Complexes with Anticancer Activity

One of the new strategies here presented concerns the combination of two different metal centres in one single drug to build a heteronuclear metal complex. The combination of more than one metal was already used for other types of applications, such as catalysis, where electronically different metals could synergistically cooperate and have better properties compared to the monometallic precursors in a catalytic process [15]. The same approach was used more recently for potential anticancer applications. The opportunity to use different metal-based compounds, which act as anticancer but have different mechanisms of action from each other, could promote cooperative activity and synergistic effects. In this review, we divided different examples into two main groups, one of platinum and one of gold. These two metal centres, as monometallic compounds, have been extensively studied and they are known to exploit their anticancer activity in different types of final targets. Platinum compounds generally act in the genomic moiety [16], while gold complexes are considered good inhibitors of thioredoxin reductase [17]. One of the most recent and interesting results of the literature concerns the combination of platinum or gold with different metal centres or a combination of them. A discussion on the possible synergistic effects of the new heteronuclear metal drugs has been done. To be sure that the enhanced anticancer activity is truly done by the cooperative action of the two metal centres, a comparison with the monometallic fragments is always needed.

### 2.1. The Case of Platinum

Transition metal complexes have been deeply studied for different applications, such as catalysis, antimicrobial, and also antitumoral drugs [18]. Platinum complexes are probably the most well-known and studied in the field of anticancer compounds, also thanks to the omnipresence of cisplatin and its derivatives as a starting point [19,20]. To overcome all the side effects of cisplatin such as neurotoxicity, nephro-, oto-, and gastrointestinal toxicity, alternative transition metal centres gained a place in this field [21,22,23]. In the last years, there are many examples of Pt(II) complexes combined with different metal centres such as Ru(II), Rh(III), Au(I), Re(I), Tc(I) [17,24,25,26,27]. Platinum-based complexes are mainly binders of genomic targets via a covalent bond between the Pt metal centre and N-7 atom of guanine base [16], even if there is also evidence of protein binding, as serum albumin [28]. Here we present the most recent and promising results of bimetallic complexes that involve different types of compounds such as photoactivable, macrocycle, or prodrugs compounds. The possible synergistic effects of two metal centres with different targets were evaluated.

Starting from the ruthenium and rhodium ones, these metal centres can form organometallic complexes usually by the coordination to nitrogen atoms or with arene ligands, making the coordination easily achievable [29]. Ruthenium-based compounds, usually considered to have antimetastatic activity, received interest as anticancer agents after the discovery of the antitumoral properties of NAMI-A, KP1019, and KP1339 mentioned above. These organometallic Ru(II)-arene derivatives have a different mechanism of action compared to cisplatin to exploit the anticancer activity, which involves the binding to the biomolecule transferrin, an iron-binding protein [30].

**Figure 1:**
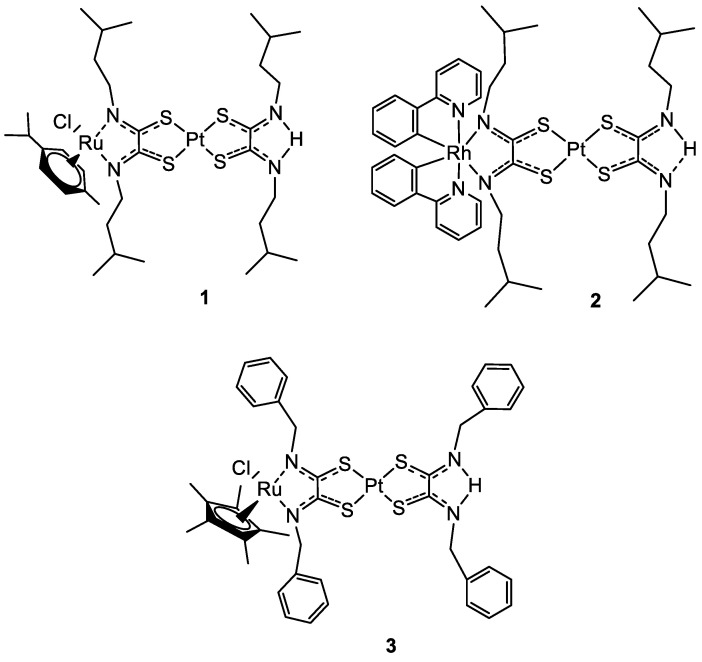
Molecular structure of compounds **1**–**3**.

In 2019 Askari and co-workers published their work, where the synthesis of three heterobimetallic Pt(II)-Ru(II) and Pt(II)-Rh(III) complexes (**1**–**3**) (Figure 1) is described. The three bimetallic complexes **1**–**3** turned out to be active in the human 20S proteasome, one of the main targets for cancer therapy. Compounds **1**–**3** also inhibited cathepsin B and L, which take part in tumour progression and invasion. The cytotoxic activity has been evaluated also in comparison to the Pt(II)-based precursors and cisplatin. The bimetallic complexes showed a good reduction of cell viability in different tumour cell lines such as neuroblastoma SH-SY5Y, melanoma SKMel-28, hepatocellular adenocarcinoma HepG2, and colorectal adenocarcinoma Caco-2 tumour cell lines, comparable or in some cases even greater than cisplatin, while for the healthy cell line of human fibroblasts WI 38 there is no sign of cytotoxicity. The flow cytometry analyses with Annexin-V/PI staining revealed that the cell death mechanism in the SH-SY5Y line is apoptotic after 72 h of treatment. Pt(II) precursors showed no significant inhibition or antiproliferative activity, which makes them a structural feature of the proposed heterobimetallic complexes [25].

**Figure 2:**
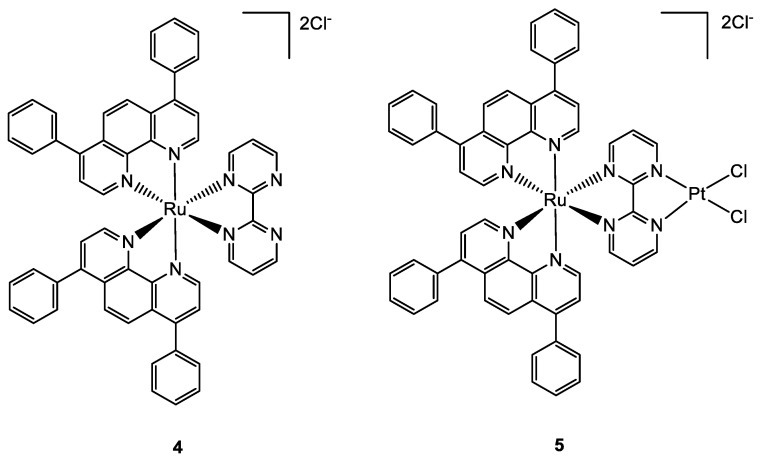
Molecular structure of compounds **4** and **5**.

**Figure 3:**
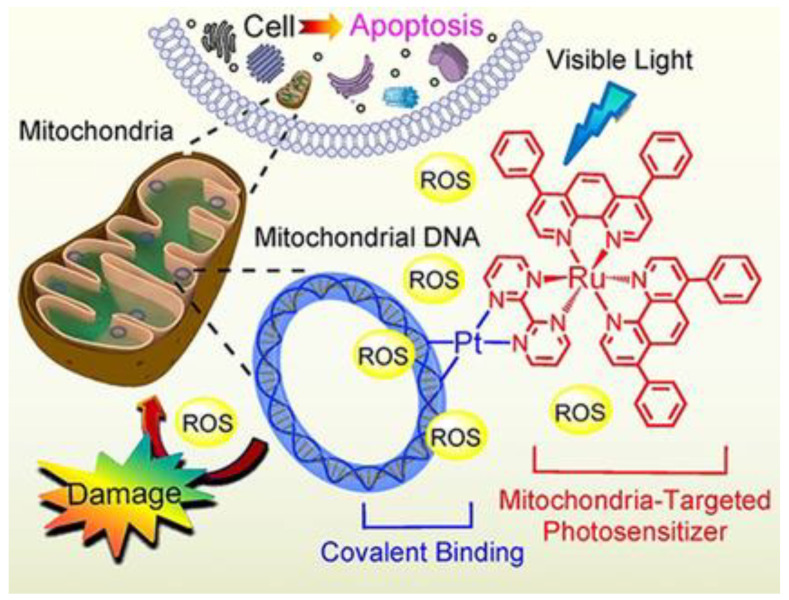
Representation of the mechanism of action of complex 5. (Reprinted with permission from ref. [31]. Copyright 2018 John Wiley and Sons).

**Figure 4:**
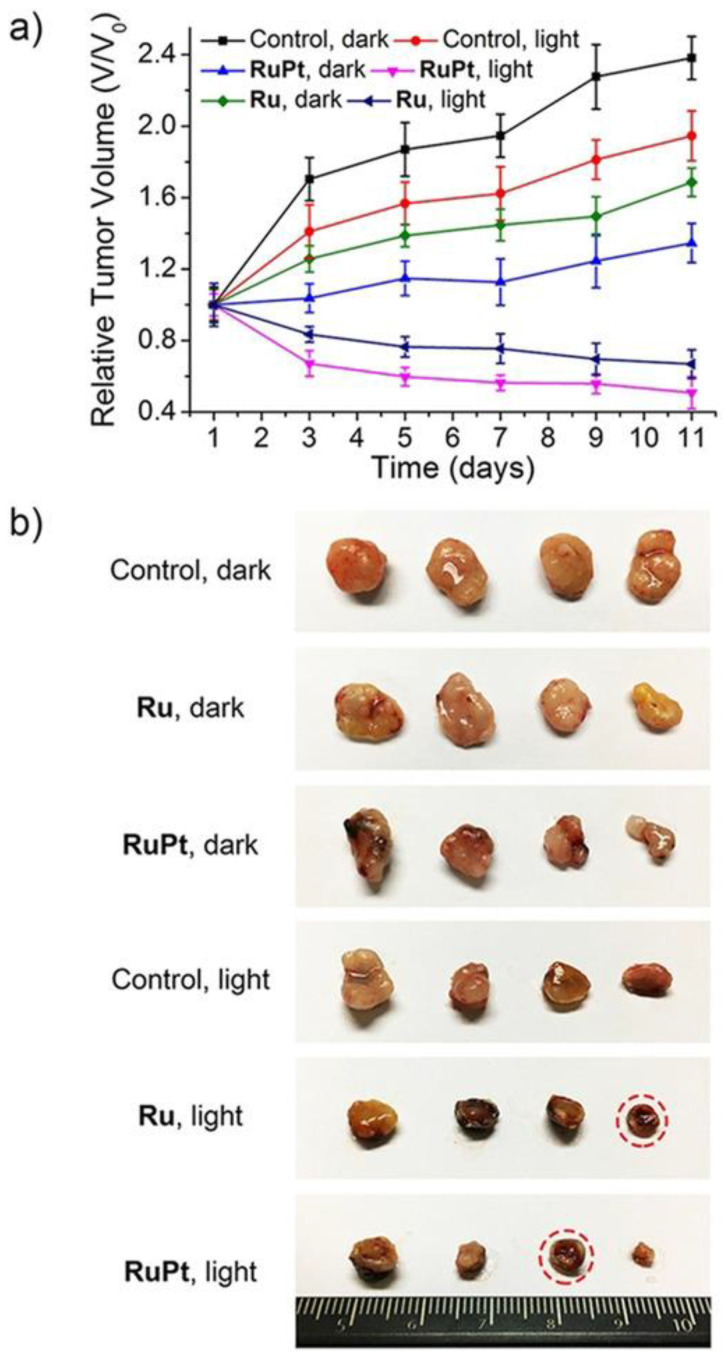
(**a**) Chart of the relative tumour volumes (V/V_0_) of mice treated with PBS, **4** (Ru), and **5** (RuPt), measured for 11 days after treatment with PBS (control), under dark and light conditions. On the first and sixth days, intratumoral injections were performed. (**b**) Photo of the tumours removed from the nude mice. The completed scavenged tumours have been underlined by red dotted circles. (Reprinted with permission from ref. [31]. Copyright 2018 John Wiley and Sons).

A paper by Zheng and co-workers, published in 2018, described the synthesis and characterization of a new bimetallic Ru(II)-Pt(II) complex (Figure 2). In this case, the platinum moiety plays a pivotal role in the activity of the whole bimetallic compound improving the anticancer activity of the Ru-containing moiety. The photoactivable Ru complex **4** is combined with cis-Pt(DMSO)_2_Cl_2_ to obtain the heterobimetallic complex **5** through the coordination of Pt to the two free nitrogen of the bpm (2,2′-bipyrimidine) ligand. The dichloroPt(II)-containing portion could covalently bind DNA. All the tests have been performed both in dark and light conditions. The cytotoxic activity of **5** is extremely increased in light conditions and is more cytotoxic compared to **4**. These results pointed out that both complexes have overcome the cisplatin resistance of the A549R (lung cancer) cell line taking advantage of the photodamage of mtDNA. Moreover, the bimetallic complex **5** can bind to mtDNA also through the Pt centre; in fact, the impairment of the polynucleotide occurs both in the dark and upon visible light irradiation. The higher affinity of **5** to DNA is due to the double type of binding: covalent, through the Pt(II) moiety, and noncovalent, through the planar ligand of Ru(II) moiety. Under light irradiation, **5** can produce ROS inside the mitochondria, causing their dysfunction, including MMP collapse, ATP depletion, and attenuation of mitochondrial energy status (see Figure 3). Both complexes have been tested in vivo and compound **5** has shown better pharmacological properties with respect to compound **4**. In particular, **5** produced lower side effects and decreased the tumour volume without causing mouse death (Figure 4) [31].

**Figure 5:**
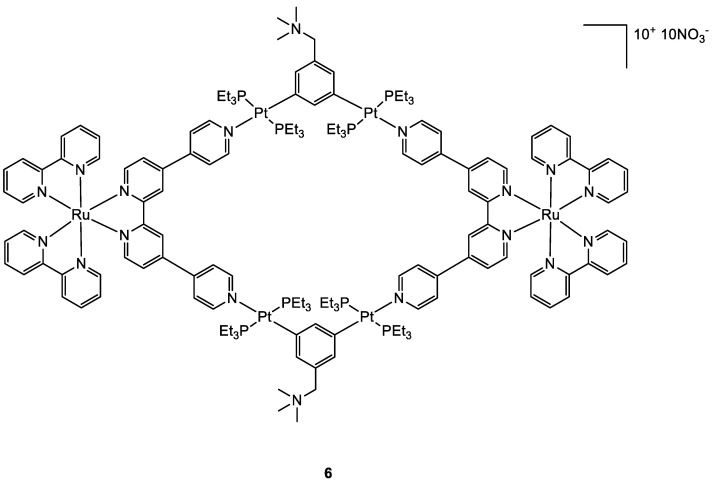
Molecular structure of compound **6**.

Photoactivable systems seem to be a good alternative to overcome cisplatin toxicity and another promising new metal complex that is active upon irradiation inside the tumour cells has been studied by Zhou and co-workers [32]. The Ru–Pt complex **6** (Figure 5) has a large macrocyclic structure, and the metallacycle facilitates its cellular uptake, as confirmed by ICP-MS analyses. The uptake values for complex **6** reach 114.82 ng and 451.37 ng per million cells for Ru and Pt, while the same test with the Ru moiety alone revealed a maximum cellular Ru concentration of 2.32 ng per million cells. The study of the cytotoxic activity on the A549 cell line showed that **6** is accumulated preferentially in mitochondria and nuclei as an intact metallacycle (confirmed by confocal laser scanning microscopy analysis). Compound **6** exhibits its cytotoxic potential upon LED irradiation at 450 nm (21.8 mW cm^−2^, 5 min), with IC_50_ values of 0.71–4.4 μM, while it is extremely decreased in dark conditions, with IC_50_ of 65.2–80.9 μM. Light promotes the generation of singlet oxygen specie which causes cell death producing damages concurrently in the mitochondria and nuclei. As already observed for products **5** and **4**, also in this case the in vivo tests at low light doses obtained with a diode laser (800 nm, 50 mW, 20 s/mm), showed that **6** causes an effective tumour remission associated with low systemic toxicity.

Another interesting strategy, that involves in this case the platinum moiety, is to exploit the advantage related to Pt(IV) complexes. The platinum centre in its higher oxidation state is chemically less reactive and kinetically more inert than its respective Pt(II) counterpart. This feature can be fruitfully used to prevent off-target reactions, obtaining complexes that can be considered prodrugs [33,34]. Moreover, the Pt(IV) complexes adopt the octahedral coordinative geometry, giving the opportunity to functionalise the axial positions with other biologically active molecules [35,36]. Since inside the cancer cells there is an increased reductive environment compared to the healthy ones [37], this can be exploited to obtain the selective Pt(IV) reduction inside the cancer cell, restoring the reactive Pt(II) complex with the simultaneous release of the axial ligands [38].

**Figure 6:**
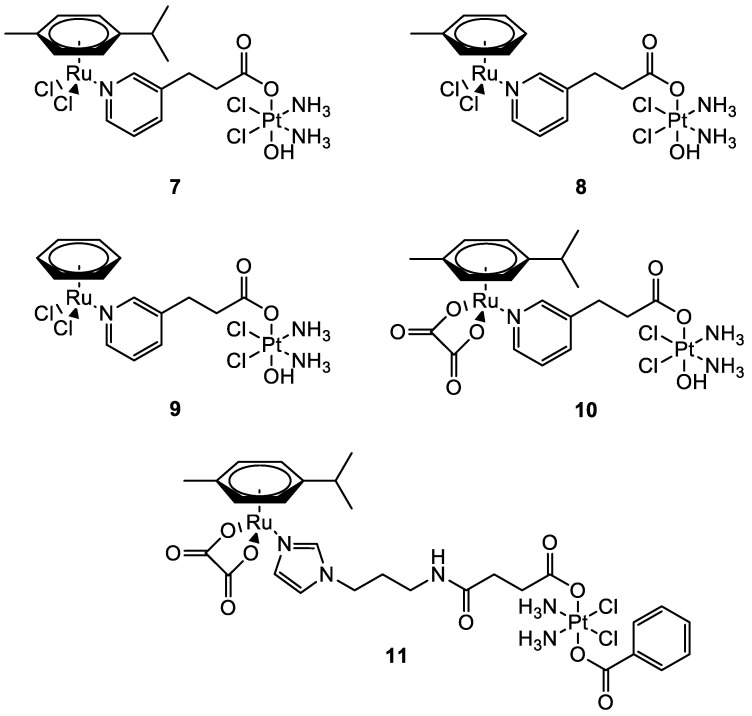
Molecular structure of compounds **7**–**11**.

In this frame, the Guangyu Zhu group worked on the synthesis of new Pt(IV)–Ru(II) metal complexes (Figure 6) which combine the cytotoxic properties of cisplatin with the antimetastatic properties of ruthenium-arene complexes. They were all found to be more cytotoxic compared to cisplatin with low micromolar (**11**), sub-micromolar, and nanomolar (**7**–**10**) cytotoxicity in most of the human cancer cells tested. All the bimetallic complexes are effective also in cisplatin-resistant cells A2780cisR (ovarian carcinoma) and A549cisR (lung adenocarcinoma). Compounds **7**–**11** have a selectivity index (the ratio between the IC_50_ in MRC-5 (lung fibroblast) and the IC_50_ in A549 cells) that is nearly 10-fold higher than that of cisplatin. A direct comparison with the performances of the monometallic moieties revealed that the increased cytotoxic activity is produced by a synergistic effect between the two metal complexes [39,40].

**Figure 7:**
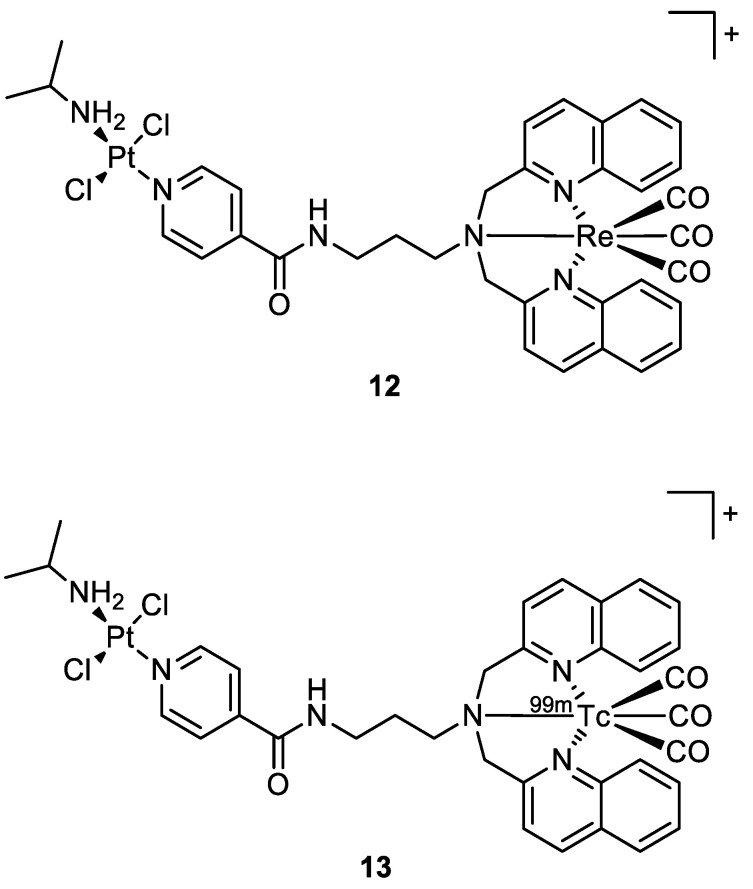
Molecular structure of compounds **12** and **13**.

Quental and co-workers investigated the combination of two different metal centres: one with therapeutic properties (i.e., Pt(II)), and the other with properties useful in diagnostics (i.e., Re(I)) to achieve a theranostic agent. The new heterobimetallic complex (**12**) (Figure 7) had indeed a rhenium moiety with photosensitizing properties and a non-classical platinum centre with intrinsic cytotoxic activity in the dark. The metalation of cellular DNA caused after the treatment with **12** both in the dark or under irradiation was investigated by employing ICP-MS analysis. After the administration of **12** in dark conditions, the polynucleotide was extracted from the cell culture and mineralised, and ICP-MS detected no presence of platinum. This agrees with gel electrophoresis experiments, where there is no modification of the mobility of the supercoiled form. On the contrary, light exposure of the cell culture treated with the studied compound increases the amount of open circular form, which is correlated to photosensitization with consequent ROS production (singlet oxygen ^1^O_2_).

The Re-tricarbonyl unit in **12** could be replaced by ^99m^Tc (**13**) (Figure 7) to allow biodistribution investigations and imaging studies in normal mice. The intensity of radioactivity was higher in the excretory organs (liver and kidney), where **13** accumulated the most [27].

**Figure 8:**
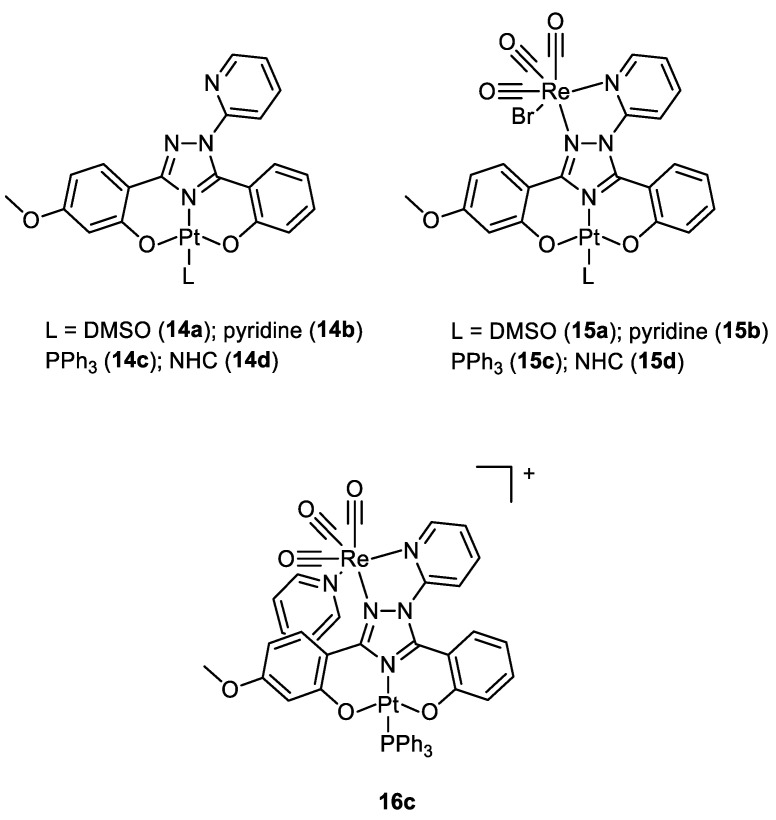
Molecular structure of compounds **14a**–**d**, **15a**–**d**, and **16c**.

In 2020, Bertrand and co-workers synthesised different bimetallic complexes of Pt(II)-Re(I) (**15a**–**d**, **16c**) starting from the Pt(II) precursors (**14a**–**d**) (Figure 8). The first screening was based on the MTT assay (Table 1). The most promising ones were the monometallic **14a** and the bimetallic **15a**, **15d,** and **16c** which were 10 times more cytotoxic compared to cisplatin. Additionally, **15b** and **15c** had no cytotoxic activity, while **16c** was the most cytotoxic of this group. Even those that showed good IC_50_ values on MDA-MB-231, MCF-7, and A2780 cancer cell lines all showed a very scarce selectivity when comparing the data obtained with the control healthy cells (MCF-10A). In addition, the lipophilicity of the whole complex (Table 1) was directly correlated to the ligand nature, and the bimetallic compounds **15a**–**d** were more lipophilic than the mono Pt counterparts, while the charged **16c** was the most hydrophilic. The amount of metal content revealed in cellular uptake experiments exactly reflected this order of lipophilicity, and it was not correlated to the cytotoxic activity. Additionally, in this case, there was no evidence of synergistic effects [24].

### 2.2. The Case of Gold

Other types of combinations involve gold compounds. Gold complexes are usually composed by the coordination of the soft metal centre with phosphine or sulphur ligands, but carbenes ligands have also gained much interest [42,43]. Gold complexes express anticancer activity by disrupting the reduction/oxidation (redox) system within the cell via the inhibition of thioredoxin reductases (TrxRs). This enzyme is present in the mitochondria and an alteration of its activity could lead to apoptosis [17,44,45,46,47]. Here we present some relevant examples of recently synthesised complexes that combine the cytotoxic potential of gold-based compounds with other metal centres which exploit their anticancer activity differently, such as Pt(II), Ru(II), Ti(IV), Ir(III), and Re(I).

**Figure 9:**
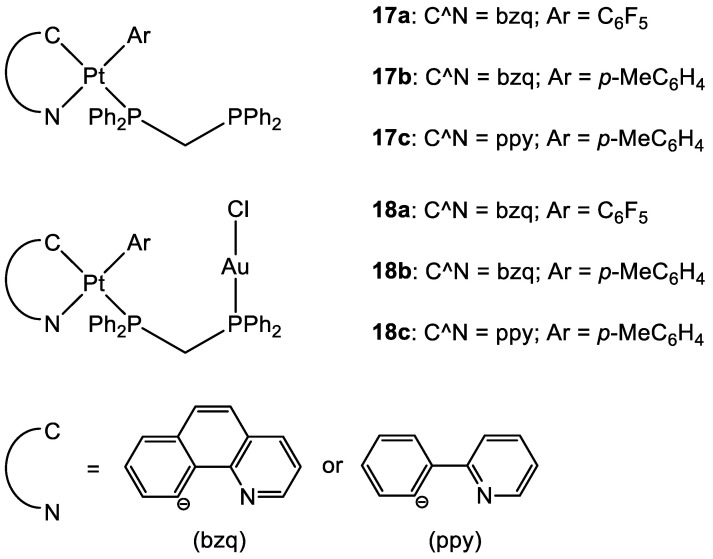
Molecular structure of compounds **17a**–**c** and **18a**–**c**.

To date, there are only a few examples in the literature on the combination of Au(I) and Pt(II) metal centres [13,48,49]. Some of the most promising examples are the heterometallic Pt–Au complexes depicted in Figure 9 (**18a**–**c**), which have shown synergistic effects by an improvement of the antiproliferative activity against A549 (lung), SKOV3 (ovarian), and MCF-7 (breast) cancer cell lines in comparison to their Pt-based precursor complexes **17a**–**c** and [ClAu(μ-dppm)AuCl], which are not toxic to any of the cell lines investigated. Inhibition of cell proliferation was also compared to cisplatin and Auranofin, revealing **18b** as the most promising complex, and apoptosis is the cell death mechanism in MCF-7 cancer cells in a dose-dependent manner. Furthermore, **18a** effectively internalises in MCF-7 cells, and the nucleus showed the highest accumulation of the complex, which was revealed by fluorescence microscopy, with less dispersion in the cytoplasm. Selectivity for cancer cells was investigated by comparing the IC_50_ values of the healthy cell line MCF-10A (epithelial breast), with the one obtained with MCF-7. All the complexes showed good selectivity for the tumorigenic cell line, and complexes **18b** and **18c** demonstrated higher specificity for human breast cancer cells with minor damage to normal epithelial breast cells [13].

**Figure 10:**
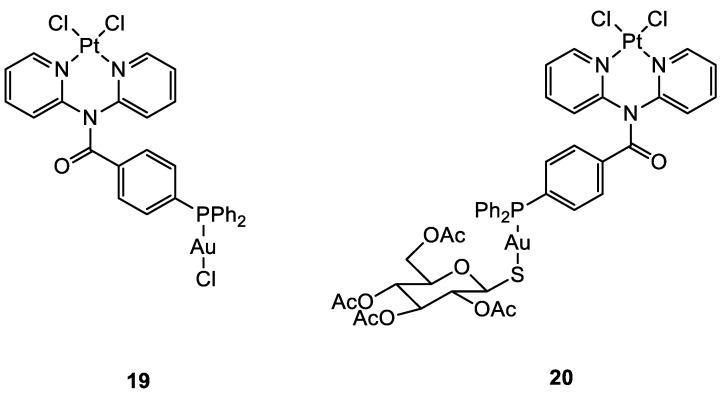
Molecular structure of compounds **19** and **20**.

In the case of the two Pt(II)-Au(I) complexes (**19, 20**) (Figure 10) proposed by Wenzel and co-workers, the new compounds exhibited great cytotoxic activity. A comparison of the IC_50_ values between the ovarian cancer cell A2780 and cisplatin-resistant A2780cis showed that **19** and **20** were able to overcome possible resistance phenomena. There is no evidence of cooperative effects of the two metals, and the cytotoxicity towards a healthy cell line is comparable to the tumoral one, demonstrating a total lack of selectivity. The sugar moiety seems to improve the transport of the compound into the cells, but the phosphine ligand may counter the Pt moiety from freely binding to the double strand of DNA [49].

**Figure 11:**
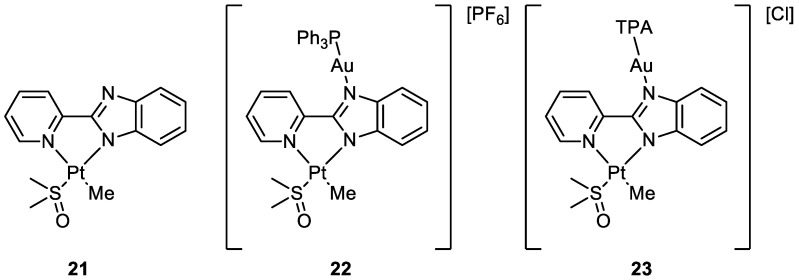
Molecular structure of compounds **21**–**23**.

Cinellu and co-workers compared the activity of monometallic Pt(II) complex **21** with the bimetallic Pt(II)-Au(I) complexes **22** and **23** (Figure 11). Interestingly, the heterobimetallic complex bearing the triphenylphosphine ligand (**22**) presented greater cytotoxic effects compared to the mononuclear Pt complex **21**. The IC_50_ value obtained after the administration of **21** and the gold(I) precursor (AF) together in an equal amount was compared with the values obtained with the heterobimetallic complexes. The data suggested an additive rather than a synergistic effect of the platinum and gold centres in compound **21**. On the other hand, it is important that even if joined in the same molecular scaffold, the two metal complexes do not reduce their biological activity. Further, the Au(TPA) ligand decreases the antiproliferative activity of complex **23**. Moreover, compounds **21** and **22** interact with RNase A but in a different way. It seems that Rnase A shows selectivity for gold over platinum. In addition, complex **22** breakdowns in the presence of the oligonucleotide (ODN4: 5′-CGC-GCG-3′), forming different adducts with the pt fragment, [AuPPh_3_]^+^ and Au(I). The formation of different biomolecular adducts, which could be caused by the presence of different metal centres in the same molecule, may lead to enhanced cytotoxic effects in agreement with the observed additive effect of coupled metals [48].

**Figure 12:**
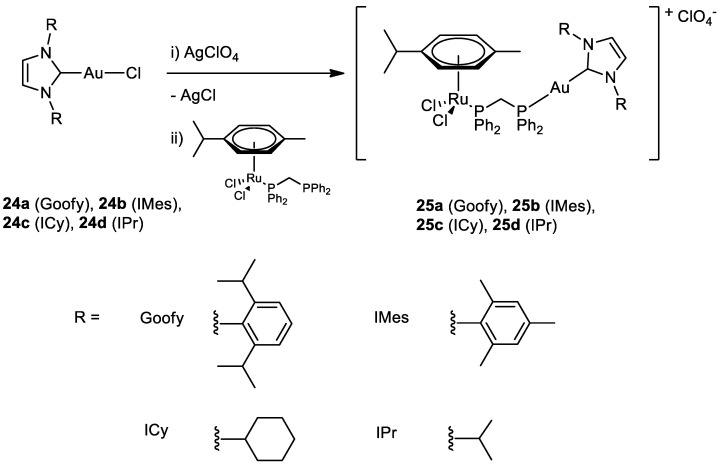
Molecular structure of compounds **24a**–**d** and **25a**–**d**.

**Figure 13:**
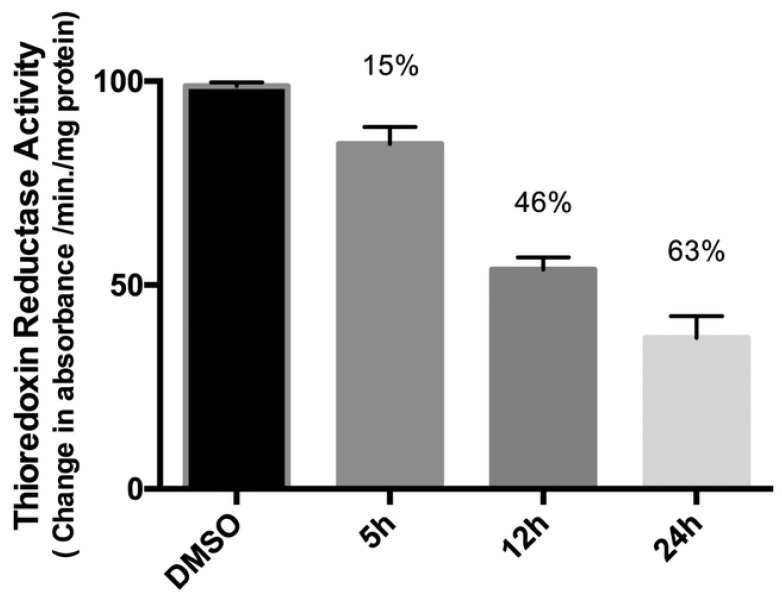
Activity of thioredoxin reductase of Caki-1 cells treated with 0.1% of DMSO (control) or complex **25a** (5 µM) after 5, 12, and 24 h of incubation. (Reprinted with permission from ref. [51]. Copyright 2016 Royal Society of Chemistry).

The group of Maria Contel synthesised new heterobimetallic Ru(II)-Au(I) complexes (**25a**–**d**) (Figure 12) where the Au metal centre is coordinated to an N-heterocycle carbene. PrestoBlue assay, a resazurin-based compound which has similar behaviour to the most well-known MTT, was used to evaluate the antiproliferative activity of the complexes [50]. The bimetallic complexes are more cytotoxic compared to cisplatin and have a better selectivity toward cancer cell lines compared to the healthy ones (HEK293T). The same assay was performed with a combination of a ratio of 1:1 of the monometallic gold (**24a**–**d**) and ruthenium counterparts and they all have a lower IC_50_ value compared to the corresponding heterometallic complexes. Compounds **25a**–**d** do not interact with the DNA plasmid pBR322, so other targets may cause cell death. Complex **25a** has been tested for the inhibition of the TrxR (thioredoxin reductase) and it is more efficient than its monometallic counterparts (Figure 13). This behaviour could suggest a synergist effect of the two metal centres when present in the same compound [51]. Further inhibition studies on complex **25b** published two years after revealed as inhibited molecular targets different interleukins, metalloproteases, and cathepsins, which are important in tumour metastasis and angiogenesis [52].

**Figure 14:**
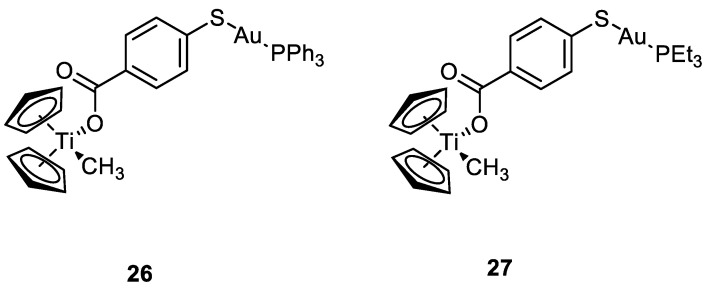
Molecular structure of compounds **26** and **27**.

The same group worked also with heterobimetallic complexes of Ti(IV)-Au(I) (**26** and **27**) (Figure 14). The mechanism of action of **26** and **27** is different compared to cisplatin, because they do not bind DNA, but they inhibit protein kinases such as p90-RSK, AKT, MAPKAPA, and thioredoxin reductase [53,54]. The two new compounds show IC_50_ values under the µM range, after 72 h of incubation, for renal cancer cell line Caki-1. Compounds **26** and **27** are more cytotoxic than the Au(I) monometallic derivatives and much more cytotoxic than the titanocene dichloride, with effective anti-migration and anti-invasive properties, making these bimetallic compounds two promising candidates for cancer therapy [55].

Ir(III) complexes can form emissive phosphorescent compounds with tuneable photophysical properties. By changing the organic ligands, the wavelength of emission changes accordingly, obtaining highly pure primary colours for OLEDs (full-colour displays), or near-infrared emission for chemical sensors, bio-imaging, and telecommunication [56,57]. Only very recently have Ir(III) complexes exhibited their potency as theranostic agents. Their emission properties enable the visualisation of their cellular biodistribution and, concomitantly, Ir(III) complexes have displayed good cytotoxicity towards different cancer cells [58,59].

**Figure 15:**
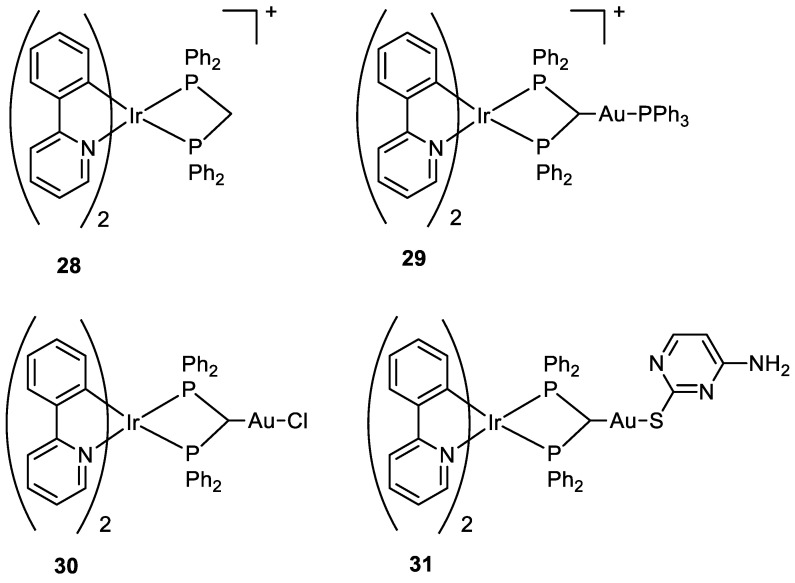
Molecular structure of compounds **28**–**31**.

Three new Au(I)-Ir(III) bimetallic complexes (**29, 30,** and **31**) (Figure 15) were presented by Redrado and co-workers in 2021 and the results were compared with the monometallic precursor [Ir(N^C)_2_(dppm)]^+^ (**28**). All complexes induce apoptosis with the formation of cytoplasmic vacuolisation. The value of IC_50_ in A549 cells ranges between 0.6 and 1.7 µM, with complex **31** being the most cytotoxic. The emission properties of Ir(III) moiety (green irradiation) allowed the investigation of cell distribution, which showed the complexes more located in the cytoplasm, and the superimposition with MitoTracker Red (MTR) emission (red irradiation), a mitochondrial selective dye used as internal standard, suggested an accumulation in the mitochondria. The presence of the gold fragment increases the generation of ROS and the inhibition of TrxR but there is no evidence of synergistic or additive effects for the heterometallic complexes [60].

**Figure 16:**
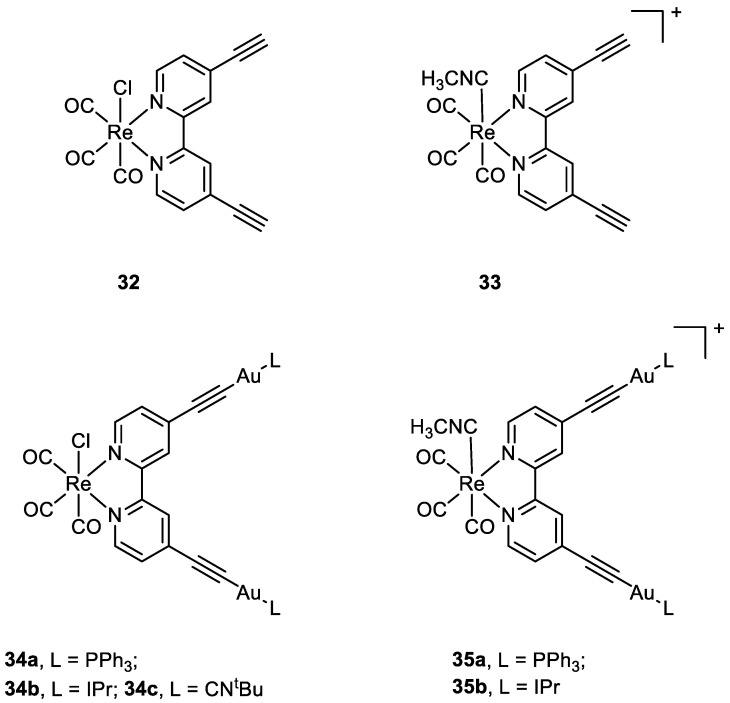
Molecular structure of compounds **32**, **33**, **34a**–**c**, and **35a**–**b**.

Luengo and co-workers published a paper on Au(I)-Re(I) heterometallic complexes (**34a**–**c** and **35a**–**b**) (Figure 16). The antiproliferative activity has been evaluated with lung cancer cells A549 and cervix cancer cells He-La. Better results have been obtained for the cationic complexes compared to the neutral ones. The monometallic Re(I) complexes (**32, 33**) show their cytotoxic activity even at 24 h, while only the heterotrimetallic complexes reveal their selectivity towards HeLa cells after 72 h with exception of **34b**. The gold moiety is involved in the mechanism of action, even in terms of internalisation. The gold ancillary ligand guides the cellular uptake following the order -PPh_3_>-CN^t^Bu>-Ipr. The distribution of the heterometallic species in HeLa cells was evaluated with fluorescent microscopy, thanks to the optical properties of Re moiety. In A549 cells all the compounds precipitate and accumulate outside the cells, especially the neutral complexes **32** and **34a**–**c**, while in HeLa cells, the positively charged heterotrimetallic compounds **35a**–**b** were in the outer cellular membrane media, very close to the membrane, suggesting a possible interaction of the complexes with the membrane. These different behaviours may explain the selectivity and higher toxicity toward the HeLa cell line over A549. The monometallic Re complex **33** internalised in the cells and no precipitate was detected outside the cell membrane. This suggests that the gold moiety could be responsible for the localisation pattern, additionally with the selectivity for HeLa cells membrane over A549 one, displayed by heterometallic species [61].

## 3. Targeting Strategies

**Figure 17:**
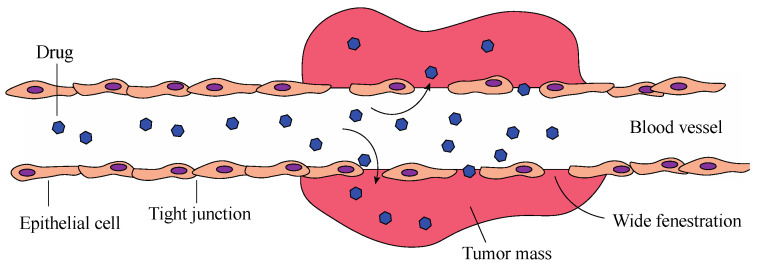
Schematic representation of the EPR effect.

In order to selectively accumulate metal complexes within tumour cells, two main approaches are generally used. The first is a sort of “passive targeting”, where the EPR effect (Enhanced Permeability and Retention) is exploited (Figure 17) [62]. Solid tumours have a particular microenvironment characterised by rich vascularization and low lymphatic drainage, where the leaky blood vessels present several wider fenestrations than healthy tissues [63]. While these characteristics allow the tumour to satisfy the augmented demand for nutrients necessary to support their rapid proliferation, at the same time, they allow drugs to have preferential extravasation into tumoral tissues [64]. However, they are not able to access so readily to healthy tissues, where the junctions between epithelial wall cells are tighter [64]. Anti-cancer nanomedicine is associated with finding delivery solutions, such as micelles, liposomes, and nanoparticles, that deliver the drug to tumour cells, mainly by exploiting the EPR effect [64,65]. Nevertheless, limited results have been achieved through passive targeting, due to the high heterogeneity of the tumour microenvironment, which can differ depending on the specific kind of tumour and its different stages [66]. In fact, tumours have a highly heterogeneous nature, and the intensity and nature of the EPR effect can vary [67]. Furthermore, this effect also depends on the chemical and physical characteristics of the drug, such as size, shape, and elasticity [68,69].

The second approach concerns using an “active targeting strategy” that can be designed according to different methods. One of the most common approaches is the use of ligands that selectively recognise biomarkers overexpressed on tumour cells, such as receptors on the cell surface or proteins in the intracellular environment [70,71]. Otherwise, other strategies foresee the use of a prodrug, inactive in healthy cells but activated only at the site of the tumour, exploiting the unique characteristics of the tumoral microenvironment, such as the presence of high levels of ROS, low pH value, overexpression of matrix metalloprotease, or the reducing conditions [72]. There are three levels towards which active drug targeting can be directed: first-order drug targeting to a specific organ or tissue, second-order drug targeting to specific cells, and third-order drug targeting to an intracellular organelle, such as the nucleus, mitochondria, endoplasmic reticulum [73,74]. This section does not intend to make a complete and exhaustive review of all cancer-targeting metal complexes. Instead, it aims to propose an overview of some of the most significant examples of methods that have been rationally designed to obtain a preferential interaction with cancer cells, according to different active targeting strategies belonging to second and third-order types.

### 3.1. Organelle–Targeting Metal Complexes

**Figure 18:**
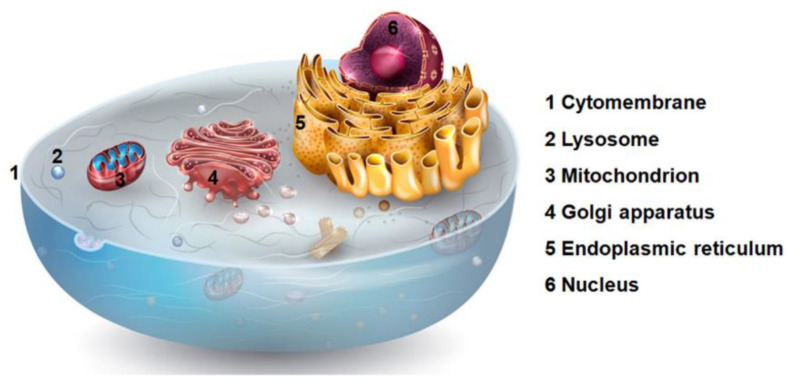
Representation of the most important organelles in the eukaryotic cell. (Reprinted with permission from ref. [76]. Copyright 2019 Elsevier).

Each organelle in the eukaryotic cell (Figure 18) plays a defined role, and for each organelle, a malfunction can lead to various diseases, including cancer [75]. This latter is one of the reasons why some organelles, such as mitochondria, have proved to be promising targets in anticancer therapy [76]; others are only recently emerging, such as the endoplasmic reticulum [77].

Organelle targeting can be achieved with metal complexes through two main strategies: coupling the complex to specific organelle-targeting biomolecules or finetuning of the complex’s properties to satisfy the characteristics required to target that particular organelle [76].

#### 3.1.1. Metal Complexes Targeting the Mitochondrion

**Figure 19:**
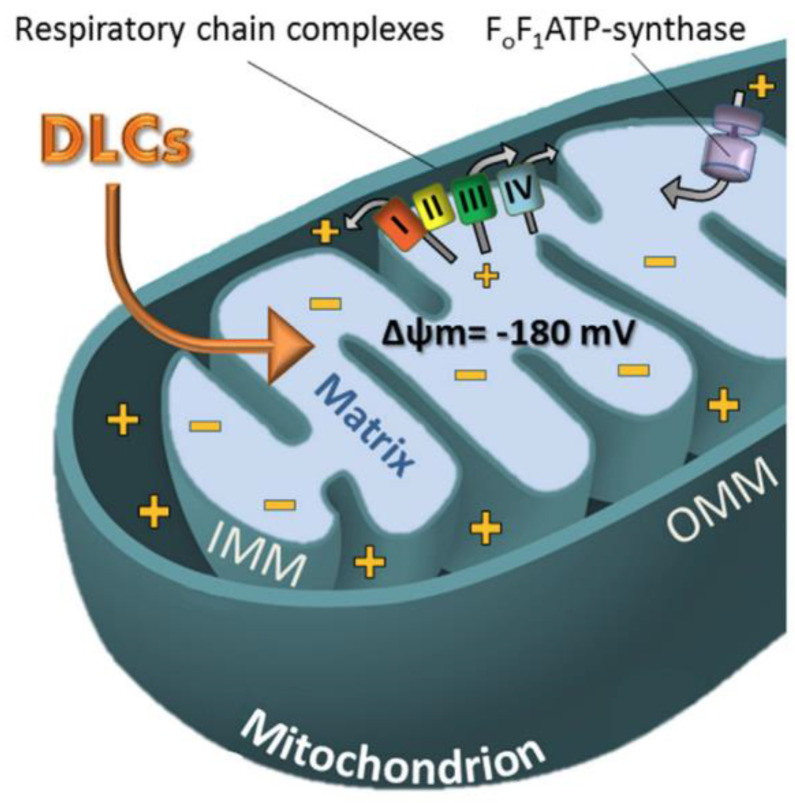
Uptake mechanism of DLCs in the mitochondrion. (Δψm: mitochondrial membrane potential; I, II, III, IV: complexes related to the mitochondrial respiratory chain; IMM: inner mitochondrial membrane; OMM: outer mitochondrial membrane). (Reprinted with permission from ref. [85]. Copyright 2018 Elsevier).

Since discovering the critical role of mitochondria in apoptosis evasion mechanisms, these organelles have attracted much interest as potential targets in anticancer therapy [78]. Mitochondria are also called the “powerhouse of the cell” because of their role in cell energy production. However, mitochondria have many other essential roles, such as biosynthetic metabolism, regulation of cell signalling, ROS neutralization through antioxidant pathways activation, adaptive functions in response to oxidative stress, and cell death induction [79]. For these reasons, mitochondria happen to be strictly connected to many different tumorigenesis pathways [80]. Deregulation of mitochondria metabolism leads to insensitivity to anti-growth signals and consequent apoptosis evasion and uncontrolled proliferation [80]. Additionally, dysfunctions in the phenomena of mitochondrial biogenesis and mitophagy, aimed at maintaining a healthy mass turnover, seem to be involved in the activations of oncogenic pathways [80]. Therapeutic strategies that rely on mitochondrial targeting have several advantages. Among the most interesting is the opportunity to exploit the difference between the structural characteristics of the mitochondrion in healthy and diseased cells to have a drug accumulation in tumour cells. Compared to normal cells, mitochondria have different characteristics in cancer cells that can be exploited in targeting strategies to direct drugs, especially towards the tumour site. For example, the increased mitochondrial transmembrane potential (Δψm), which has been highlighted in many kinds of cancer, can be exploited to selectively accumulate delocalised lipophilic cations (DLCs) within mitochondria (Figure 19) [81,82]. This selectivity depends on the DLCs chemical-physical characteristics, especially the positive charge number and the lipophilicity (logP) tuning [83,84].

In addition, considering the central role of this organelle in tumorigenesis and in sustaining cancer cell proliferation, drugs that accumulate within the mitochondrion can inhibit its functions, cause its impairment, and activate proapoptotic signalling pathways leading to programmed cell death [86]. Beyond the issue of poor selectivity, mitochondrial targeting drugs may potentially overcome another recurring problem in classic anticancer therapy such as drug resistance. Cisplatin-induced resistance seems to depend on the action of the nucleotide excision repair (NER) machinery, which repairs alterations in DNA that prevent replication [87]. This repair pathway is only active in the nucleus while it is absent in the mitochondrion; for this reason, it is far more difficult that the damage induced by cisplatin on the mitochondrial DNA leads the onset of resistance [87]. Erxleben published an extensive review in 2019 in which 241 references regarding mitochondrion targeting metal complexes were reported [86].

In the present review, this section provides some of the most significant examples of mitochondria-targeting complexes divided by the type of metal centre.

(1). Platinum-based complexes targeting mitochondria

**Figure 20:**
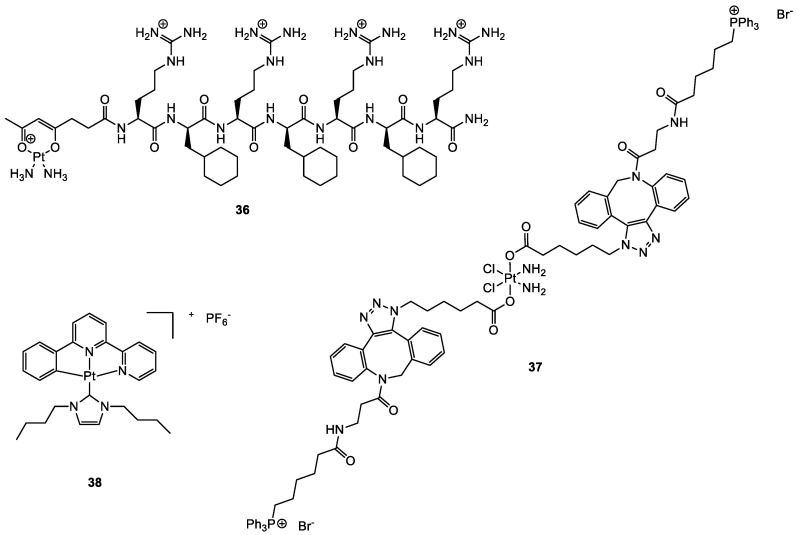
Structure of complexes **36**–**38**.

Although the primary target for platinum-based complexes is nuclear DNA, some studies have shown that cisplatin may have interesting direct interactions with mitochondrial DNA (mtDNA), therefore inducting apoptosis also through different mechanisms, in addition to the expected ones or also alternatively [87,88,89]. Intending to redirect cisplatin onto mitochondrial DNA, Wisnovsky et al. synthesised a cisplatin analogue (mtPt, **36**; Figure 20) conjugated to a peptide carrier that explicitly targets this organelle [88]. mtPt shows accumulation in the mitochondrion, cytotoxicity on HeLa cells, and damage to mtDNA without damaging nuclear DNA. Although cytotoxicity is lower than cisplatin, mtPt is also efficient in killing cisplatin-resistant ovarian A2780/CP70 cancer cells.

**Figure 21:**
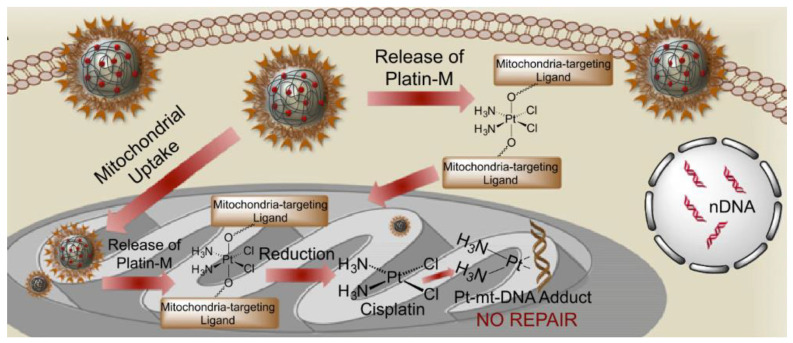
Mechanism of the release of Platin-M. (Reprinted from ref. [87] under the CC BY-NC-ND license, 2014).

Dhar and collaborators used a different carrier to deliver cisplatin selectively into mitochondria [87]. They designed a cisplatin Pt(IV) prodrug that targets these organelles, Platin-M (**37**, Figure 20). Mitochondrial targeting is provided by the presence of two delocalised triphenylphosphonium cations on the Pt(IVI) complex in the axial position. Drug delivery, good pharmacokinetic, and distribution properties are achieved using biocompatible polymeric nanoparticles (NP) constituted by biodegradable poly(lactic-co-glycolic acid)-block-polyethyleneglycol, functionalised with a terminal triphenylphosphonium cation too, obtaining in this way a double targeting effect. Compared to cisplatin alone, Platin-M NPs resulted in a 30-times higher Pt concentration in mitochondria than in the nucleus. Prodrug Platin-M is locally activated by the highly reducing environment and converted into the active Pt(II) cisplatin, which was observed to form cross-links with mtDNA (Figure 21). Platin-M NPs showed 85 times higher potency than cisplatin in resistant A2780/CP70 cells, and enhanced cytotoxicity was also underlined in human neuroblastoma SH-SY5Y cells and androgen-independent Pca cells. Although the insertion of TPP moiety was successful in achieving mitochondrial targeting in the case of Platin-M, this strategy is not universally applicable; in fact, other platinum-based complexes that exhibit TPP functionalization accumulate in the nucleus [90].

Che and coworkers proposed a luminescent Pt(II) complex (**38**, Figure 20), that features an N-heterocyclic carbene as a ligand [91]. Given the optimal emission properties, the cellular localization of the synthesised complex was studied through fluorescence microscopy, incubating it with fluorescent dyes such as MitoTracker^TM^, Hoechst 3342, and LysoTracker^TM^ in order to highlight the localization in mitochondria and cytoplasm, in the nucleus and lysosomes, respectively. Experiments showed that the complex is preferentially co-localised in mitochondria with MitoTracker^TM^. Complex **38** was found to show promising cytotoxicity towards HeLa, HepG2, SUNE1, and CCD-19Lu cell lines (IC_50_ = 0.057–0.77 µM), with a 300-fold higher potency toward HeLa than cisplatin.

(2). Gold-based complexes targeting mitochondria

**Figure 22:**
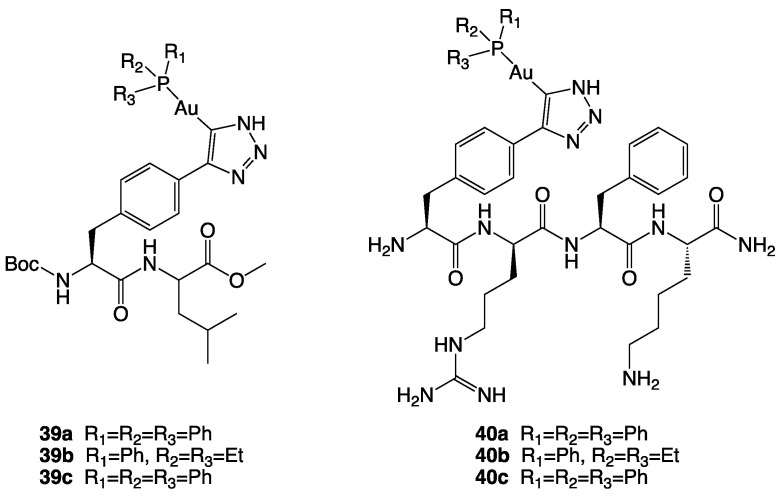
Structures of complexes **39a**–**c** and **40a**–**c**.

The thioredoxin (Trx) system, composed of thioredoxin reductase (TrxR), thioredoxin, and NADPH, plays an essential role in regulating cellular redox balance [92]. TrxR is a ubiquitous homodimeric flavoenzyme responsible for reducing several species, including the protein thioredoxin. In mammals, two principal isoforms have been isolated, TrxR-1 (cytosolic) and TrxR-2 (mitochondrial). Cancer cell often shows TrxR overexpression, especially the isoform TrxR-2, which seems to be induced by increased levels of oxidative stress [85]. In several types of tumours, TrxR-2 has an important role in the desensitization of cancer cells towards pro-apoptotic signals; in fact, it neutralises reactive oxygen species (ROS), which are considered important mediators of apoptosis [85,93]. As a result, TrxR has emerged as a potential target for anticancer therapies, and there has been a growing interest in metal complexes that can inhibit TrxR and induce apoptosis in cancer cells. Gold complexes have shown interesting cytotoxic activities on several tumour cell lines, and although the mechanism of action has not yet been fully elucidated, the main mechanism is explicated through the inhibition of mitochondrial thioredoxin reductase [94,95]. Regarding gold complexes, mitochondrial targeting has been achieved through two main methods. The first one, a second-order targeting, involves the conjugation of the gold complex to mitochondria-targeting carriers that allow the accumulation in the organelle. One example of carrier-conjugated gold complexes was synthesised by Köster and collaborators [96]. They exploited the mitochondrial targeting properties of di- and tetra-peptides, using them as carriers for the selective delivery of Au(I) phosphines [96]. The peptide-Au(I)phosphine conjugates (**39**–**40**, Figure 22) showed a cytotoxicity (2–50 µM) that seems to be correlated to their lipophilicity. This latter seems to be responsible for a higher initial uptake. Through this strategy, they could overcome the cisplatin resistance in MDA-MB231 breast cancer cells.

The second method involves a third-order targeting strategy: the use of complexes able to target mitochondrial proteins, the most studied of which is thioredoxin reductase in this context. Various metal-based complexes can inhibit thioredoxin reductase (e.g., complexes based on silver, copper, platinum, bismuth, palladium, antimony, iron, and ruthenium) [85]. However, gold-based complexes are still the most promising ones and seem to have the most significant potential for selective targeting strategies. Gold has a high affinity for selenium, present in the selenocysteine at the C-terminal end of the enzyme’s active site [95]. This latter amino acid is relatively easy to target since, with a pKa of 5.4, it has a higher nucleophilicity than cysteine and is easily accessible on the enzyme structure [97]. Although there is an overexpression of TrxR in many tumour types, the preferential affinity of gold-based compounds for TrxR is not sufficient in most cases to ensure selective cytotoxicity on diseased cells without harming healthy cells. However, there are a few examples of gold-based compounds that showed selectivity in cancer cells. For the synthesis of gold-based complexes, N-heterocyclic carbenes ligands are particularly useful in the fine control of the complexes’ lipophilicity, thanks to the structural modifications on the heterocyclic ring that can be made in a relatively easy way [98]

**Figure 23:**
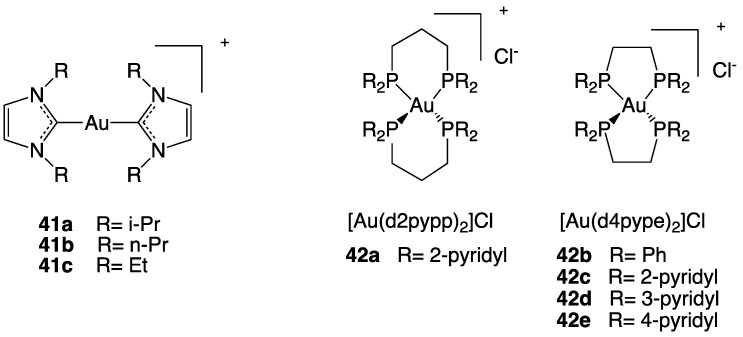
Structures of complexes **41a**–**c** and **42a**–**e**.

Hickey and collaborators designed three Au(I) N-heterocyclic carbene (Au-NHC) compounds (**41**, Figure 23) that combine TrxR inhibition and, due to their properties as DLCs, also the mitochondria targeting action [99].

**Figure 24:**
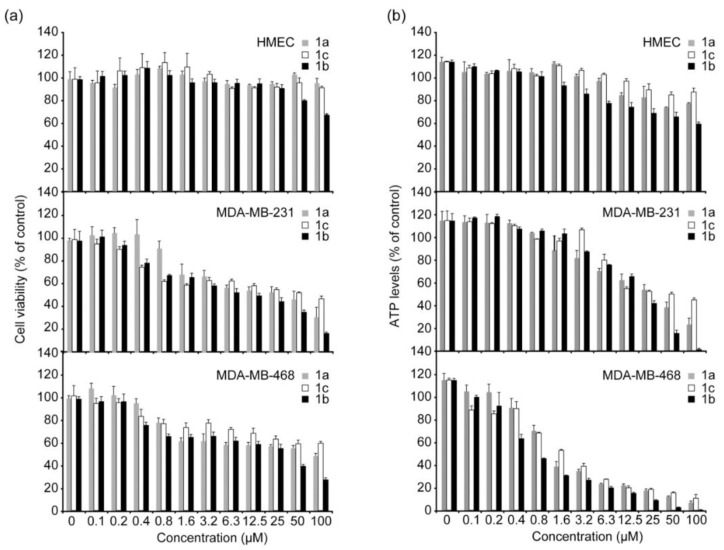
(Panel **a**) Cell growth measured 24 h after treatment with **6a**, **6b**, and **6c**. (Panel **b**) ATP levels of cells measured 24 h after treatment, using the ATPlite luminescence assay kit. (Reprinted with permission from ref. [99]. Copyright 2008 American Chemical Society).

The effects of different concentrations of the three complexes were evaluated on cell growth of MDA-MB-231 and MDA-MB-468, two breast cancer cell lines, and also on HMEC, healthy human mammary epithelial cells. The three Au(I) NHC complexes have shown selective toxicity to both cancer cell lines but not to the normal cells, and the degree of selectivity is correlated with their lipophilicity. Among the three complexes, the **41a** complex, presenting an intermediate log P value, shows the best selectivity and cytotoxic potency (Figure 24).

In addition to carbenes, phosphines can also be used as ligands to obtain gold complexes having DLCs properties, able to target the mitochondrion. A study on the anticancer activity of eight bis-chelated Au(I) bidentate phosphine complexes was reported by Rackham and collaborators [100] that revealed selective anti-cancer properties of the complex [Au(d2-pypp)_2_]Cl, presenting 1,3-bis(di-2-pyridylphosphino)propane as ligand (**42**, Figure 23). This gold complex selectively induces apoptosis in breast cancer cells MDA-MB-468 but not in normal breast cells HMEC, at submicromolar concentrations (0.8 μM). Gold complex’s accumulation in mitochondria is driven by the characteristic cancer cells’ high potential difference; thus, apoptosis induction is accomplished through the mitochondrial pathway, involving mitochondrial membrane potential depolarization, depletion of the glutathione pool, and caspase-3 and caspase-9 activation.

In general, gold complexes are not able to discriminate between the two TrxR isoforms. However, there are some examples of selective inhibition of TrxR-2 [85]. The two isoforms have a high percentage of identity, and it is therefore rather difficult to achieve selectivity on either of them. However, as the two isoforms are distributed in different cell compartments, a promising way to achieve a selective inhibition of TrxR-2 is to use the mitochondrial targeting strategy so that the accumulated drug can preferentially interact with the mitochondrial isoform.

(3). Ruthenium-based complexes targeting mitochondria

**Figure 25:**
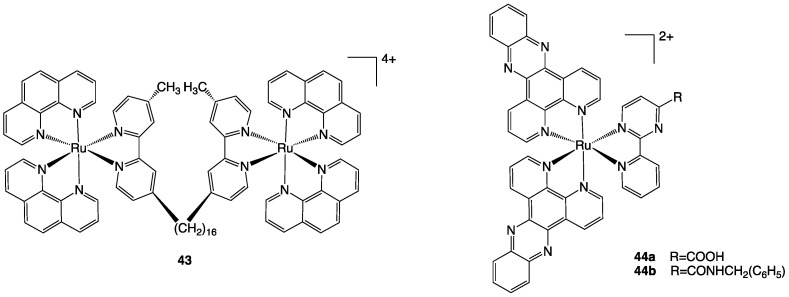
Structures of complexes **43** and **44a**–**b**.

Ruthenium-based complexes show promising cytotoxic activity against various cancer cell lines, including cisplatin-resistant ones, due to their ability to interact with DNA and RNA [101]. Although their primary target is generally nuclear genomic material, some examples of DLCs Ru(II) polypyridyl complexes accumulate in the mitochondrion, where they also exert their cytotoxic action [102]. A significant example is Rubb_16_ (**43**, Figure 25), a dinuclear complex reported by Pisani et al., which shows an IC_50_ of about 5 µM on L1210 murine leukaemia cancer cell line [103]. Even if the cytotoxicity is comparable to that of cisplatin, Rubb_16_ presents 16 times higher accumulation in tumour cells than in healthy B cells. Rubb_16_ is part of a series of lipophilic cations that present two metal centres connected by a flexible bridge, with the lipophilicity of the entire complex increasing as the number of methylenes in the linker rises. Using confocal microscopy and a mitochondrial tracking dye (MitoTracker Green FM), the cellular localization of the luminescent complex was highlighted in the mitochondrion, with no staining observed in the cytoplasm or other organelles. Interesting results emerged from the study on the two complexes **44a** and **44b** (Figure 25), which, despite having relatively similar molecular structures, have a considerable difference in cytotoxicity and cellular localization. Cytotoxicity was assessed in five cancer cell lines and one healthy cell line, human lung fibroblasts MRC-5. Complex **44a** had similar cytotoxicity to cisplatin but higher selectivity to diseased cells, while complex **44b** had a considerably higher IC_50_. Confocal microscopy studies showed that complex **44a** is localised in the cytoplasm with a weak fluorescence in the nucleus, unlike complex **44b**, which diffuses through the whole cell, including the nucleus. Then, using high-resolution continuum source atomic absorption spectrometry (HR-CS AAS), a mitochondrial uptake of 68% of the total was measured in HeLa cells for **44a**. For these reasons, although complex **44a** interacts with isolated DNA, a mechanism of action via the mitochondrial pathway has been proposed, characterised by a perturbation of the mitochondrial membrane potential caused by the intercalation of the hydrophobic dppz moieties.

Interestingly, the conjugation of **44a** to mitochondria-targeting or receptor-targeting peptides, performed by the same group, led to the lack of accumulation in mitochondria and, consequently, cytotoxic potency being compromised [104].

#### 3.1.2. Metal-Based Complexes Targeting the Nucleus

The nucleus is not only the organelle that contains most of the genetic material, but it is also referred to as the “brain” of the cell, being responsible for vital cellular functions such as replication, gene expression, and cell differentiation [105]. As nuclear DNA is one of the main targets of metal-based anticancer drugs, nuclear drug delivery is crucial for optimal therapeutic efficiency. The drugs transport from the cytoplasm to the nucleus through the nuclear envelope lipidic bilayer does not take place by passive diffusion but via the nuclear pore complex (NPC), a cylindrical structure of nucleoporins that forms a hydrophilic channel through which the bidirectional transport of macromolecules between the nucleus and cytoplasm takes place [106]. Regarding NPCs, diffusion pores with an estimated diameter of 90–100 Å allow free transit of macromolecules up to 40 kDa, whereas large macromolecules with a diameter of up to 390 Å require an active transport mediated by specific receptors [107]. Nucleus-targeting metal complexes can be obtained either intrinsically or extrinsically. Intrinsic nuclear targeting complexes usually have positive charges, allowing electrostatic interaction with the phosphate groups in DNA and a planar aromatic system allowing intercalation in the double helix [76]. Conversely, extrinsic nucleus-targeting activity can be achieved by conjugating the metal complex to a nuclear localization signal (NLS), a short peptide that selectively binds the nuclear transport receptors (NTRs), that facilitate the passage across the NPCs central channel [76].

**Figure 26:**
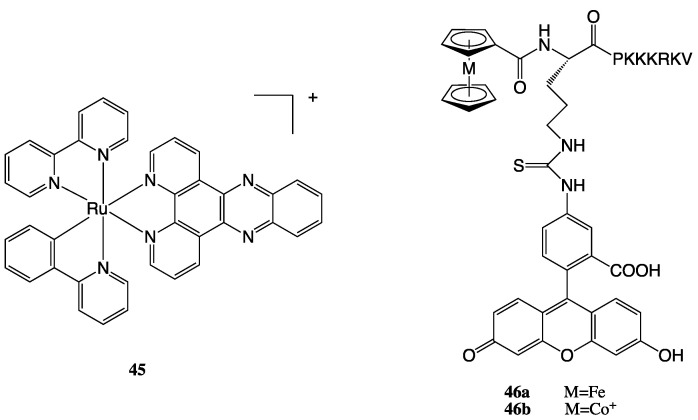
Structures of complexes **45** and **46a**–**b**.

**Figure 27:**
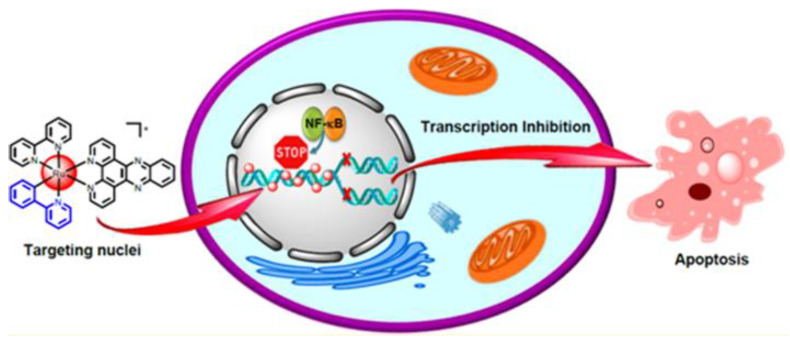
Representation of [Ru(bpy)(phpy)(dppz)]^+^ nucleus-targeting activity. (Reprinted with permission from ref. [108]. Copyright 2014 American Chemical Society).

An interesting example of intrinsic targeting is [Ru(bpy)(phpy)(dppz)]^+^ (**45**, Figure 26) [108], a Ru(II) complex that showed rapid uptake in Hela cells, with nearly 90% of the complex accumulating in the nuclei. It showed a similar distribution also in MDA-MB-231, A549, and A549/CDDP cancer cell lines, with an IC_50_ value that was an order of magnitude lower than cisplatin. Further research indicated that the strong DNA binding affinity of [Ru(bpy)(phpy)(dppz)]^+^ effectively inhibited the binding of NF-B transcription factor to DNA sequences, causing cancer cell apoptosis (Figure 27).

Concerning extrinsic targeting, Noor et al. synthesised a bioconjugate where an organometallic complex, a cobaltocenium cation, was linked to an NLS peptide, SV-40 T (with primary sequence H-Pro-Lys-Lys-Lys-Arg-Lys-Val-OH) [109]. The conjugate shows enhanced cellular uptake and a significant accumulation in the nucleus of HepG2 cells. The same group studied the uptake and cellular localization of some neutral ferrocene (Fe^2+^) and positively charged Cobaltocenium (Co^3+^) bioconjugates (**46**, Figure 26) [110], where the metal-based core was conjugated with the NLS wild-type sequence PKKKRKV. Although they did not find the complexes cytotoxic on the Hep G2 cell line, for both metal centres the fluorescence microscopy study showed significant cellular uptake and nuclear localization of the conjugates. Interestingly, the results obtained are similar for the two metal centres, thus highlighting that the positive charge may not appear decisive in nuclear targeting.

#### 3.1.3. Metal-Based Complexes Targeting Endoplasmic Reticulum

It has been demonstrated that the endoplasmic reticulum (ER) is not just passively responsible for the transport of proteins in the intracellular or extracellular space, but it also plays a key role in regulating cellular homeostasis; in fact, the insurgence of ER stress seems to be closely related to the onset of pathological states, such as neurodegenerative disorders, diabetes, cardiac diseases, and cancer [111]. Features usually present in the tumour environment, such as low pH, lack of oxygen, strongly reducing environment, and lack of nutrients, can lead to perturbations of protein homeostasis, inducing ER stress and consequently activating signalling transduction pathways such as the unfolded protein response (UPR) [112]. This latter is an adaptive mechanism contributing to cancer growth, aggressiveness, and resistance to the treatment. For this reason, ER has attracted much interest as a promising target to address potential anticancer drugs according to a selective anticancer strategy. Notwithstanding the UPR being a key element in cancer growth and chemotherapy resistance, the promotion of ER stress induction has lately received attention as a promising anticancer technique. In particular, certain metal complexes are able to induce ER stress, which often leads to desirable immunogenic cell death. Additionally, an interesting aspect is that ER-targeting cytotoxic compounds often accomplish selectivity on cancer cells [77]. Drugs can induce ER stress through different mechanisms, such as direct contact with UPR machinery, generation of ROS, disruption of protein folding chaperones, inhibition of protein degradation, and interference with Ca^2+^ trafficking [77]. Metal complexes capable of generating ER stress in cancer cells through various mechanisms include complexes based on copper [113,114], vanadium [115], iridium [116,117], ruthenium [118,119], gold [120,121], platinum [122], palladium [123], osmium [124,125], and rhenium [126].

**Figure 28:**
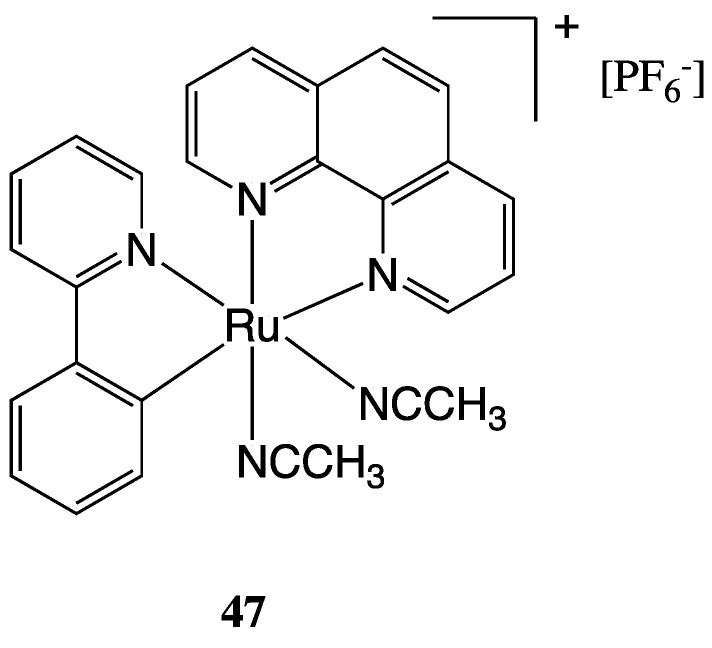
Structure of compound **47**.

**Figure 29:**
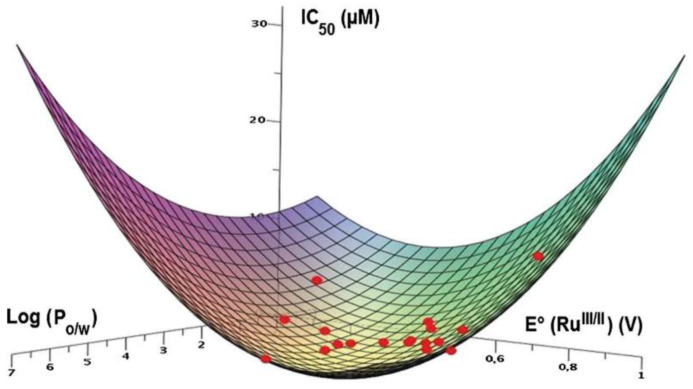
Three-dimensional representation of the correlation between IC_50_, the redox potential (E°_RuIII/II_) and the lipophilicity (logP_o/w_). (Reprinted with permission from ref. [118]. Copyright 2011 Royal Society of Chemistry).

However, it is essential to emphasise that several mechanisms are frequently activated at the same time, and furthermore, activation of ER stress is often not the primary mechanism of action of a metal complex but rather a collateral mechanism. A promising investigated class of compounds with ER stress-inducing activities is cyclometalated cationic Ru(II) complexes. In this context, Fetzer et al. reported an interesting study on a library of 32 organoruthenium C-N, N-C-N, N-N-C cyclometalated compounds that have been synthesised and tested for their in vitro antitumoral activity on HCT-116 colon cancer cell lines [118]. The library of compounds originates from the original lead compound RDC11 (**47**), whose structure is reported in Figure 28. Good to excellent cytotoxic properties have been found for the complexes, many of which have demonstrated an IC_50_ below the nanomolar threshold. The compounds’ activity was correlated to their physicochemical properties, such as the Ru^III/II^ redox potential and lipophilicity, as shown in Figure 29. Further investigation of the biological properties and cellular responses reveals that some compounds cause both DNA damage and ER stress, while others only target the ER [119].

### 3.2. Cell-Targeting Metal-Based Complexes

**Figure 30:**
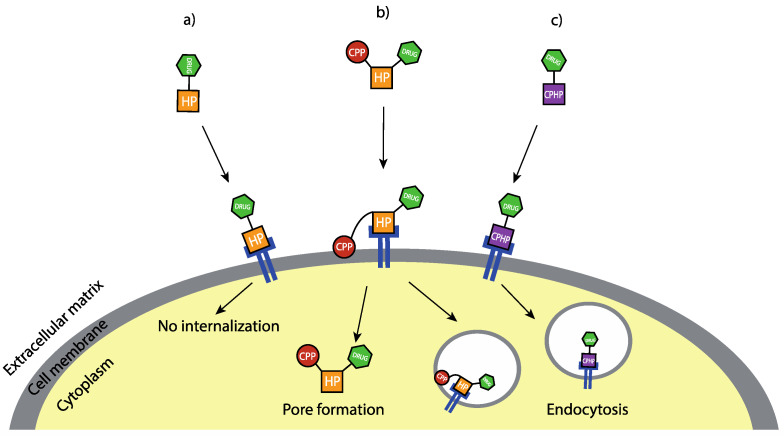
A representation of the three mechanisms of action of HP (**a**), CPP linked to an HP (**b**), CPHP (**c**).

In the human organism, cells deputed to different functions or belonging to different tissues present a heterogeneity expressed by specific biomarkers existing on the cell surface, often consisting of proteins and receptors. The same also applies to diseased cells, such as cancer cells, that usually show a cancer-specific overexpression of particular receptors [127]. In order to specifically target only cancer cells exploiting these cancer-specific biomarkers, cytotoxic compounds can be conjugated to selected ligands capable of being recognised and bound by receptors overexpressed on the cancer cell surface and then internalised via endocytosis, then released into the cytoplasm [128]. Peptides, antibodies, aptamers, or natural ligands of cell receptors can represent valuable site-selective tools to distinguish between cancer and healthy cells based on cellular surface different compositions. The most widely used and studied ligands for metal complexes delivery are the cell-penetrating homing peptides (CPHPs) [129,130]. It is necessary to specify that there are various types of peptides capable of delivering the drug to the cell, and the mode and ability to reach the cell’s interior vary greatly. Homing peptides (HP) can recognise the corresponding receptor and bind it but are not internalised within the cell, remaining on the membrane surface in the extracellular environment [129,130]. In contrast, cell-penetrating homing peptides (CPHPs) can bind the receptor and have the intrinsic ability to activate the internalization process through endocytosis. Another technique used is binding an HP, responsible for the recognition, to a cell-penetrating peptide (CPP), responsible for activating the internalization process (Figure 30) [128].

**Figure 31:**
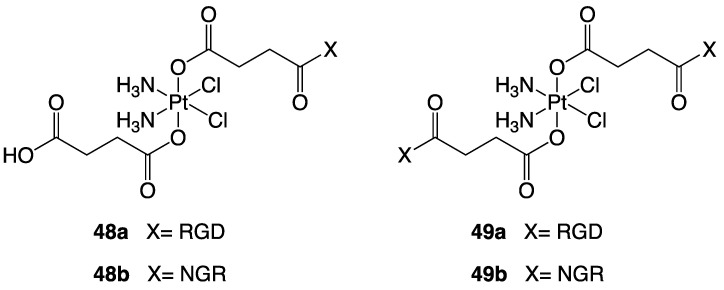
Structures of compounds **48a**–**b** and **49a**–**b**.

Lippard and his team reported in 2007 the synthesis of a series of mono- and di-functionalised platinum(IV) complexes (**48**–**49**, Figure 31) conjugated to an RGD or NGR peptides [131]. These latter target α_V_β_3_/ α_V_β_5_ integrins and membrane-spanning surface protein aminopeptidase N (APN), respectively, both of which are over-expressed in endothelial cells of angiogenic tumour vasculature. The HP has been conjugated by an amide linkage to the Pt(IV) centre through a succinate group. From concentration-response studies emerged that, when compared to nontargeting Pt(IV) compounds and the unconjugated targeting RGD peptide, RGD-conjugated Pt(IV) complexes are potent inhibitors of cellular growth. Although less inhibitive than their RGD counterparts, NGR conjugates were nonetheless more active than nonspecific Pt(IV)-peptide analogues.

As mentioned above, in addition to peptides, other kinds of site-selective cancer-targeting molecules can also be conjugated to metal complexes to obtain a targeting activity on specific cancer cell lines. An interesting example is that of aptamers, which are short single-stranded chains of oligonucleotides that can specifically bind their targets with high affinity, as well as being biocompatible and stable [132].

**Figure 32:**
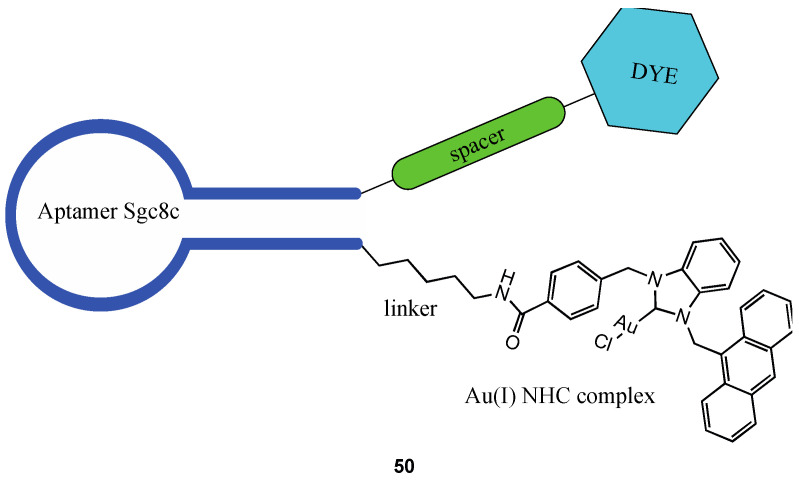
Structure of the bioconjugate **50**.

Niu et al. reported the synthesis of a bioconjugate (**50**, Figure 32) in which an NHC gold(I) complex has been linked to the sgc8c aptamer, which can selectively recognise CCRF-CEM leukaemia cells binding the protein tyrosine kinase 7(PTK-7), more abundantly expressed in cancer cells surface than in healthy ones [133].

The bioconjugate is enriched with a double tag, making it possible to independently follow the fate of the two functional elements inside the cancer cell. An anthracenyl moiety was inserted to follow the gold complex, while the aptamer was functionalised with fluorescein isothiocyanate (FITC). Remarkably, the IC_50_ of the aptamer-functionalised complex shows higher cytotoxicity than the non-targeting complex, going from a value of 14.6 ± 1.4 µM to 0.54 ± 0.85 µM.

## 4. Conclusions

Inspired by the promising anticancer properties of some well-known metal-based complexes, such as auranofin, cisplatin, and NAMI, new strategies have been developed to improve the characteristics of the existing complexes and overcome the problems that they carry. Higher selectivity, lower side effects, higher potency, and the overcoming of the resistance mechanism are some of the objectives of this new generation of anticancer strategies. In this review, we focused on some interesting examples related to two main approaches: the conjugation of metal-based complexes to a targeting moiety and the synthesis of heterobimetallic complexes bearing two active molecules in just one compound. We believe this review can be a valuable tool to be used as a starting point for those researching this area, to identify the recent advances that have been made in research and to exploit the competencies to progress towards new ideas and strategies.

## Figures and Tables

**Table 1:** Antiproliferative activity of compounds **14a**, **15a**, **15d**, **16c**, and cisplatin on different cancer cell lines and the healthy cell line MCF-10F after 72 h of incubation at 37 °C. Partition coefficients of all complexes are reported as Log P_o/w_. (Adapted with permission from ref. [24]. Copyright 2020 John Wiley and Sons).

Complex	IC_50_ ± SD (µM)				Log P_o/w_
	MDA-MB-231	MCF-7	A2780	MCF-10F	
**14a**	3.1 ± 0.5	1.6 ± 0.9	1.5 ± 0.6	1.1 ± 0.2	3.7
**14b**	n. d.	n. d.	n. d.	n. d.	3.3
**14c**	n. d.	n. d.	n. d.	n. d.	6.9
**14d**	n. d.	n. d.	n. d.	n. d.	4.5
**15a**	2.1 ± 0.6	2.2 ± 0.4	1.5 ± 0.3	1.82 ± 0.06	3.6
**15b**	n. d.	n. d.	n. d.	n. d.	4.9
**15c**	n. d.	n. d.	n. d.	n. d.	8.1
**15d**	9.2 ± 3.5	20.3 ± 1.9	34.5 ± 7.2	17.4 ± 2.6	5.8
**16c**	1.7 ± 0.3.	1.1 ± 0.4	1.3 ± 0.2	3.3 ± 0.3	2.4
cisplatin	20.4 ± 3.4	14 ± 3.5	1.0 ± 0.2	2.9 ± 0.8	−2.4 ^a^

n. d. = not determined; ^a^ data retrieved from ref [41].

## Data Availability

Not applicable.
